# Virological Surveillance of Influenza A Subtypes Isolated in 2014 from Clinical Outbreaks in Canadian Swine

**DOI:** 10.3390/v9030055

**Published:** 2017-03-21

**Authors:** Helena Grgić, Jackie Gallant, Zvonimir Poljak

**Affiliations:** 1 Department of Population Medicine, Ontario Veterinary College, University of Guelph, 50 Stone Road East, Guelph, ON N1G 2W1, Canada; 2 Centre for Public Health and Zoonoses, University of Guelph, Guelph, ON N1G 2W1, Canada; 3 Gallant Custom Laboratories, 1425 Bishop St. N Units 10-13, Cambridge, ON N1R 6J9, Canada; jackie@gallantcustomlaboratories.com

**Keywords:** influenza A virus, triple-reassortant, H3N2, H1N2, swine, genome

## Abstract

Influenza A viruses (IAVs) are respiratory pathogens associated with an acute respiratory disease that occurs year-round in swine production. It is currently one of the most important pathogens in swine populations, with the potential to infect other host species including humans. Ongoing research indicates that the three major subtypes of IAV—H1N1, H1N2, and H3N2—continue to expand in their genetic and antigenic diversity. In this study, we conducted a comprehensive genomic analysis of 16 IAVs isolated from different clinical outbreaks in Alberta, Manitoba, Ontario, and Saskatchewan in 2014. We also examined the genetic basis for probable antigenic differences among sequenced viruses. On the basis of phylogenetic analysis, all 13 Canadian H3N2 viruses belonged to cluster IV, eight H3N2 viruses were part of the IV-C cluster, and one virus belonged to the IV-B and one to the IV-D cluster. Based on standards used in this study, three H3N2 viruses could not be clearly classified into any currently established group within cluster IV (A to F). Three H1N2 viruses were part of the H1α cluster.

## 1. Introduction

Influenza A virus (IAV) infection remains one of the important causes of respiratory disease in swine populations. The first clinical report of influenza-like disease in pigs was coincident with the 1918 “Spanish flu” [1], and the causative agent was subsequently characterized as H1N1 [2]. This classical-swine H1N1 (cH1N1) remained stable and the predominant subtype for almost 80 years in North American swine populations. In the late 1990s, novel double- and triple-reassortant H3N2 (trH3N2) viruses were reported in swine populations [3]. Reassortment between seasonal human H3N2 and cH1N1 resulted in double-reassortant viruses, while additional reassortment with the avian influenza virus resulted in the trH3N2 virus [3]. These triple-reassortant viruses subsequently further reassorted with cH1N1 viruses, resulting in distinct H1N1 or H1N2 subtype lineages [4]. The H1 subtype viruses have been grouped into contemporary α-, β-, and γ- cluster viruses related to the classical H1 lineage that evolved by drift mutation and reassortment with trH3N2 viruses [5]. However, the *H1* gene of δ-cluster viruses has been derived from seasonal human H1N1 [5]. Currently, North American swine H1 and H3 influenza viruses are grouped into H1-α, -β, -γ, and -δ and H3 -I, -II, -III, and IV (A to F) clusters, based on antigenic variation [6,7]. Recently, a novel clade of H1γ-2 viruses with unique genome constellations has been detected [6]. Authors demonstrated distinct antigenic variations and differences between the novel H1γ-2 clade and others currently circulating H1γ [6].

In Canada, H1N1 had been a dominant IAV subtype in swine prior to 2005 [8] when the trH3N2 strain emerged and swiftly spread throughout the country [9]. In 2009, the H1N1 pandemic virus—A(H1N1)pdm09—emerged in swine populations, with the first case being identified in Alberta [10]. Detailed molecular studies conducted soon thereafter indicated a number of distinct H1N1 viruses, with their *HA* genes categorized as similar to αH1 cluster viruses, or to the A(H1N1)pdm09, or occasionally to seasonal human influenza virus genes [11]. In more recent studies, the A(H1N1)pdm09 has been identified as the most prevalent H1N1 virus in Ontario swine [12], and H1N2 reassortants have been documented [12,13]. Different types of novel H3N2 reassortants have been documented in several different studies [14,15], as well as ongoing circulation of the trH3N2 virus [15,16].

IAVs circulating in swine represent a considerable concern for the health management of these populations [17], but also pose a potential risk to human health due to the possibility of spillover infections, particularly if they could result in sustained chains of transmission among people. Insights into patterns of swine IAV genetic and antigenic diversity are critical to identify risks to swine and human populations for interspecies transmission and to provide criteria for updating influenza diagnostics and vaccine composition. In this study, we conducted a comprehensive genomic analysis of Canadian swine IAVs collected in 2014, and we examined the genetic basis for potential antigenic differences among sequenced viruses.

## 2. Material and Methods

### 2.1. Viruses

Viruses isolated in 2014 from outbreaks of respiratory disease in pigs (Alberta, Manitoba, Ontario, and Saskatchewan; Table 1) were obtained from the Gallant Custom Laboratories (Cambridge, ON, Canada). Viruses were propagated in Madin-Darby canine kidney (MDCK) cells as described elsewhere [18]. Harvested cell culture supernatant was clarified by centrifugation and viruses were concentrated by ultracentrifugation over a 20% sucrose cushion. Virus pellets were resuspended overnight at 4 °C in nuclease-free water and stored at −80 °C. Virus isolates were identified by a hemagglutination (HA) assay, as described elsewhere [18].

### 2.2. Genomic Sequencing and Sequence Assembly

The complete nucleotide sequences of 16 viruses were performed using the Illumina platform. Illumina Sequencing By Synthesis (SBS) was performed by the Clinical Genomics Centre, the UHN/MSH Gene Profiling Facility (Mount Sinai Hospital, Toronto, ON, Canada). Briefly, the RNA sequencing libraries were constructed using the Illumina TruSeq RNA Library Prep Kit V2 according to the manufacturer’s instructions (Illumina, San Diego, CA, USA). RNA was fragmented into pieces using divalent cations under elevated temperature, and reverse transcribed into complementary DNA (cDNA) using SuperScript II Reverse Transcriptase (Thermo Fisher Scientific, Carlsbad, CA, USA) and random primers. After end repair and ligation of the sequencing adaptors, the cDNA templates were double purified using AMPure XP beads (Beckman Coulter, Brea, CA, USA). The DNA fragments that had adapter molecules ligated on both ends were enriched with 12 cycles of PCR. Sequencing was performed with an Illumina Miseq (Illumina, San Diego, CA, USA) for 300 cycles of paired-end sequencing run. The Illumina data pipeline, including image analysis, base-calling, and quality-filtering was performed using the Illumina sequence analysis software, Casava (Version 1.8.2).

### 2.3. Sequence Analysis

To determine the gene relatedness of each gene segment of all 16 swine IAVs, we used Basic Local Alignment Search Tool (BLAST) from the Genbank database. Geneious Pro 5.5.6 has been employed to determine nucleotide and amino acid (aa) sequence identities. The CLUSTAL W alignment method was carried out, and an unrooted phylogenetic tree of the *HA*, *NA*, and *M* genes was constructed by using a Jukes–Cantor genetic distance model and the Unweighted Pair Group Method with Arithmetic Mean (UPGMA) three-building method. The *HA* genes of two subtypes were additionally analyzed by using Neighbor Joining (NJ) trees based on the distance method with 1000 bootstraps through PAUP 4 [19]; and by using 100,000 samplings with 10,000 burn-in through MrBayes [20]. To predict N-linked glycosylation sites (Asn-X-Ser/Thr, where X is any aa except Pro) we utilized the NetNGlyc 1.0 server. Genetic mapping was conducted using non-parametric multidimensional scaling using the *smacof* library [21] in R statistical software (Version 3.1).

## 3. Results

### 3.1. Genetic Characterization of IAV Isolated from Clinical Outbreaks in Canadian Swine

Here we analyzed 13 swine H3N2 and three swine H1N2 viruses isolated in 2014 from clinical outbreaks in Alberta, Manitoba, Ontario, and Saskatchewan. Structurally, the 13 swine H3N2 viruses detected in this study could be classified into five groups. The first group consisted of four viruses in which all eight segments were similar to those of the trH3N2 virus that emerged in 2005 in Ontario. The second group consisted of five viruses with *HA*, *NA*, *PB1*, and *NS* genes originating from the trH3N2 virus, and *PA*, *PB2*, *NP*, and *M* genes originating from the pandemic A(H1N1)pdm09 virus. The third group consisted of two viruses with *HA*, *NA*, *PB1*, *PB2*, *NP*, and *NS* genes originating from the trH3N2, and *PA* and *M* genes originating from the A(H1N1)pdm09 virus. The fourth group consisted of one virus with *HA*, *NA*, *PB1*, *PB2*, and *NS* genes originating from the trH3N2 virus, and *PA*, *M*, and *NP* genes originating from the A(H1N1)pdm09 virus. The fifth group consisted of one virus with *HA*, *NA*, *PBI*, *PB2*, and *PA* genes originating from the trH3N2 virus, and *NP*, *M*, and *NS* genes originating from the A(H1N1)pdm09 virus. Sequence data for the viruses in the current report have been deposited in Genbank; accession numbers of the reported sequences are KX264674-KX264791. Genome constellations identified in Canadian H3N2 and H1N2 viruses isolated from swine in 2014 are presented in Table 2.

**Figure 1:**
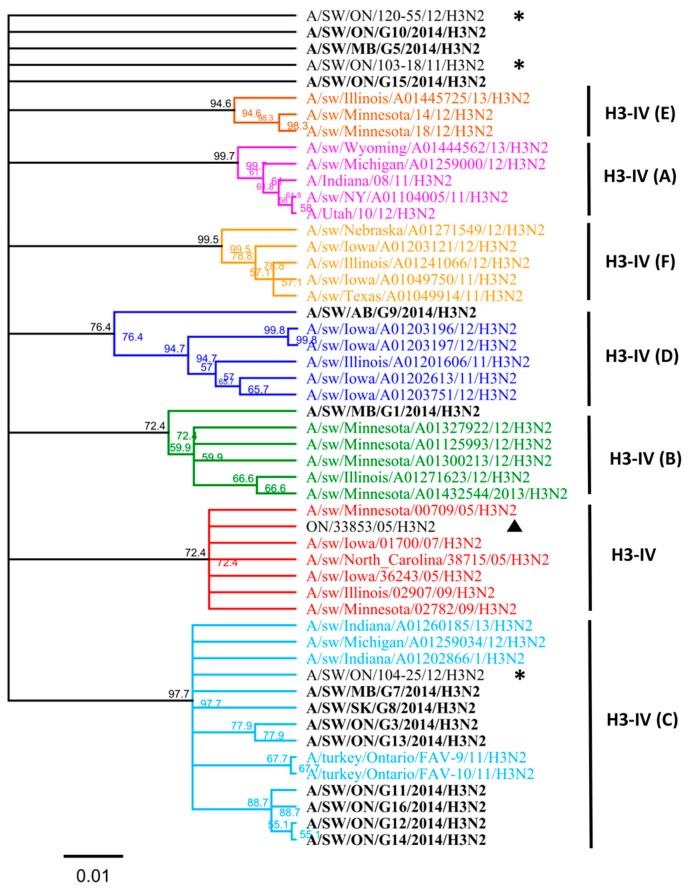
The H3N2 *HA* phylogeny of Cluster IV from A to F (indicated on right), based on amino acid (aa) sequences of the HA1 region. Thirteen Canadian H3N2 viruses isolated during 2014 from swine are labeled in black. Ontario viruses isolated in 2011–2012 are indicated by an asterisk. The 2005 Ontario virus representative of Cluster IV is marked with ▲. The scale represents the number of substitutions per site.

**Figure 2:**
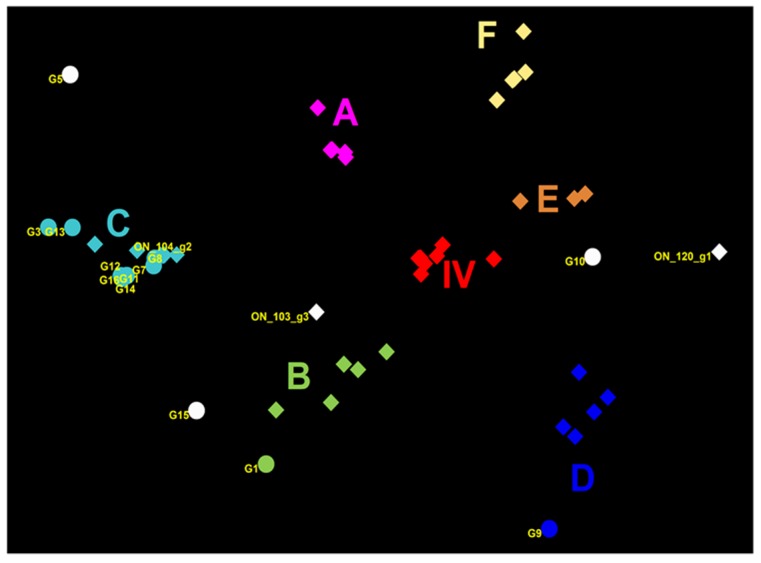
Genetic map of 13 Canadian H3N2 influenza A viruses (IAVs) and representative H3N2 viruses using the HA1 domain aa sequences. The genetic map was based on pairwise differences among strains. Viruses are color-coded according to their grouping into subclusters of Cluster IV H3N2 viruses and additionally labelled using the subcluster name (A to F). Viruses that could not be classified into any existing cluster IV are colored in white. Circles represent virus isolates from the current study, whereas rectangles represent previously detected viruses. The *ON_120_g1* is a representative of the Group 1 viruses previously detected in Ontario [15] (A/SW/ON/120-55/12/H3N2); *ON_104_g2* is a representative of Group 2 (A/SW/ON/104-25/12/H3N2), and *ON_103_g3* is a representative of the Group 3 viruses (A/SW/ON/103-18/11/H3N2) from the same study [12]. Labels represent the following study viruses: G1 = A/SW/MB/G1/2014, G3 = A/SW/ON/G3/2014, G5 = A/SW/MB/G5/2014, G7 = A/SW/MB/G7/2014, G8 = A/SW/SK/G8/2014, G9 = A/SW/AB/G9/2014, G10 = A/SW/ON/G10/2014, G11 = A/SW/ON/G11/2014, G12 = A/SW/ON/G12/2014, G13 = A/SW/ON/G13/2014, G14 = A/SW/ON/G14/2014, G15 = A/SW/ON/G15/2014, G16 = A/SW/ON/G16/2014.

Nucleotide sequence analysis of *HA* genes of the H3 subtype showed that all Canadian viruses could be grouped within cluster IV viruses. The *HA* gene of 13 Canadian H3N2 viruses showed an identity of 93.4% to 99.3% with Ontario swine viruses isolated between 2011 and 2012. Interestingly, when compared to the original Cluster IV trH3N2 (A/sw/ON/33853/05/H3N2) virus from 2005, the *HA* gene of the 13 Canadian viruses showed 95.8% to 97.2% identity. Overall, nucleotide sequence identity between the *HA* genes of the 13 H3N2 Canadian viruses analyzed in this study ranged from a minimum of 92.2% to a maximum of 99.9%. According to the previously suggested criteria of Anderson et al. [6], 5%–7% average pairwise nucleotide distance could be considered a threshold to define the new putative clusters of contemporary swine H3 viruses. Therefore, the study viruses were compared with recently formed genetic clusters identified within cluster IV (A to F), referred herein also as subclusters. Analysis based on aa sequences of the HA1 domain of H3 subtype viruses showed that eight H3N2 viruses belonged to the IV-C cluster, one H3N2 virus belonged to IV-B and IV-D clusters (Figure 1 and Figure 2). Results of the analysis, using NJ and Bayesian approaches, are available in the supplementary material and are in agreement with respect to the classification of study viruses into distinct groups (Figures S4 and S5).

Three isolates could not be clearly classified into any of the existing subclusters (A to F) when compared to standards used in this study. Thus, the aa sequences of the HA1 domain of these three H3N2 viruses (A/sw/MB/G5/14, A/SW/ON/G10/14, and A/SW/ON/G15/14) have been compared with published IAV sequences. The maximum identity of the A/SW/MB/G5/2014 isolate, based on the aa sequence of the HA1 region, was with the index H3N2 Cluster IV virus in Ontario detected in 2005 (A/sw/ON/33853/05/H3N2) and with A/sw/Minnesota/02782/09/H3N2 (93.6%), both of which have been grouped into the original cluster IV viruses (Figure 1). Still, this virus had 13 aa changes relative to the original Ontario virus (Table S1). Similar identity results were obtained when a BLAST search based on the HA1 portion was conducted (data not shown), although the identity of the entire *HA* gene was higher, at 96%, with two distinct viruses detected in Iowa and Minnesota (Table S2), both at the nucleotide and the aa level. 

The maximum identity of A/SW/ON/G10/2014 isolate, again based on the aa sequence of the HA1 region, was 96% with A/sw/ON/33853/05/H3N2 and 96.3% with A/sw/Minnesota/02782/09/H3N2. This virus had seven aa changes relative to the A/sw/ON/33853/05/H3N2 (Table S2). Similar results were obtained when a BLAST search based on the HA1 portion was conducted when a maximum identity remained 97% (data not shown), similar to the identity of the entire *HA* gene which was 97% with two distinct viruses detected in Manitoba in 2005 and 2007 (Table S2).

The maximum identity of A/SW/ON/G15/2014 isolate was 94.8% with A/sw/ON/33853/05/H3N2 and A/sw/Minnesota/02782/09/H3N2, both of which form the original cluster IV virus. This virus had 14 aa changes relative to the index trH3N2 virus in Ontario (A/sw/ON/33853/05/H3N2). However, when a BLAST search based on the HA1 portion was conducted it resulted in a maximum identity of 99.4% with viruses from Indiana and Manitoba (data not shown); which was similar to the identity of the entire *HA* determined to be 99% with two distinct viruses detected in Iowa and Indiana in 2015 and 2016 (Table S2).

**Figure 3:**
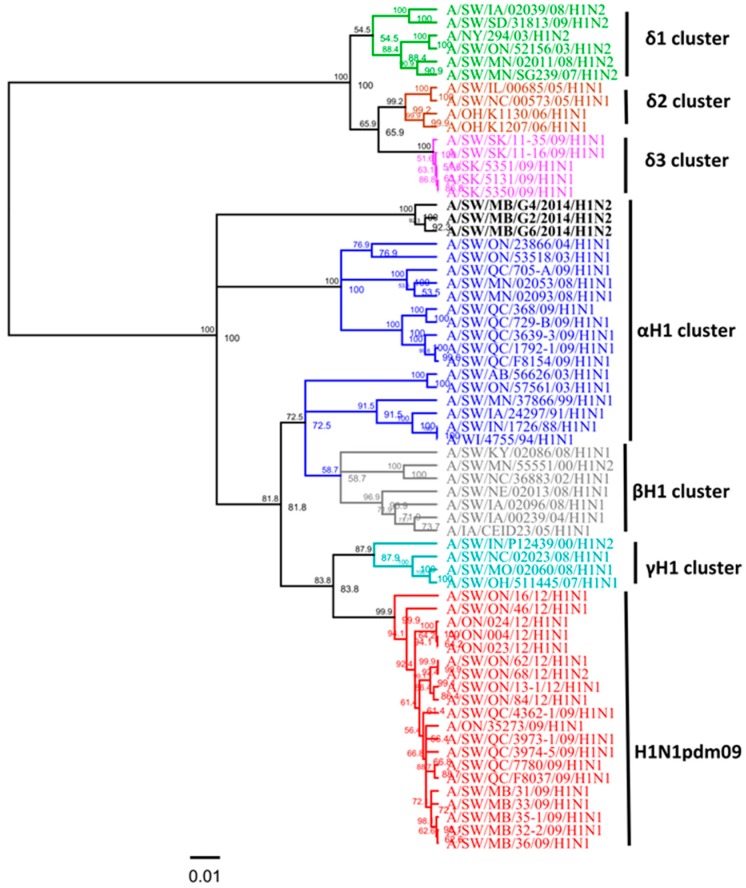
Phylogenetic analysis of the full hemagglutinin (*HA*) gene of three Canadian H1N2 viruses isolated from swine in 2014 (black) based on nucleotide sequences. IAV in swine (IAV-S) H1α (blue), β (gray), γ (turquoise), δ1 (green), δ2 (brown), δ3 (pink) clusters and pandemic (H1N1pdm09) (red) have been included in this analysis. The scale represents the number of substitutions per site.

Three swine H1N2 viruses isolated in 2014 had the *HA* gene that belongs to the αH1 cluster (Figure 3), and had *PA* and *M* genes originating from the 2009 A(H1N1)pdm09 virus and *NA*, *PB1*, *PB2*, *NP*, and *NS* genes originating from the trH3N2 virus (Table 2). Overall, nucleotide sequence identity between the *HA* genes of the three H1N2 Canadian viruses analyzed in this study ranged from 98.3% to 99.1%. However, when compared with published IAV sequences, the *HA* gene of these three H1N2 viruses (A/SW/MB/G2/14, A/SW/MB/G4/14, and A/SW/MB/G6/14) exhibited the highest identity of 99% with A/sw/MB/D0347/14/H1N2, A/sw/MB/D0339/14/H1N2, and A/sw/MB/D0296/13/H1N2, respectively. Within the αH1cluster, two distinct sub-clusters have been observed: the H1N1 alpha viruses known to previously circulate and a new cluster of H1N2 alpha viruses initially detected in Manitoba in the fall of 2013 [13]. Results of the analysis using NJ and Bayesian approaches are available in the supplementary material, with identical classification of study viruses (Figures S6 and S7). The three H1N2 viruses from this study contained a unique deletion of two aa at positions 129 and 130 in the H1 numbering system. Observed deletions of two aa were located in the receptor-binding pocket, which potentially might have an effect on the ability of the virus to bind cell surface receptors (sialic acid) and infect host cells.

**Figure 4:**
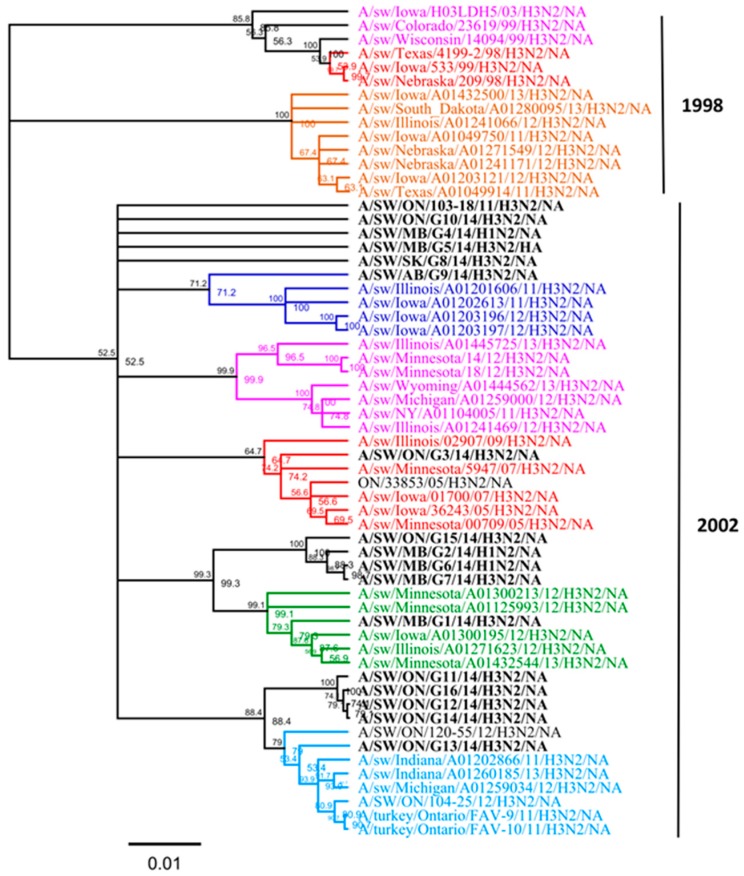
Phylogenetic analysis of the neuraminidase (*NA*) gene of 13 H3N2 and three H1N2 viruses isolated in 2014 from different clinical outbreaks in Alberta, Manitoba, Ontario, and Saskatchewan (bold black) and sequences from viruses in a previous study [15]. The branches have been colored by a *HA* genetic cluster. The H3N2 *NA* sublineages are indicated on the right side (1998 opposed to 2002). The tree is produced on the basis of nucleotide sequences. The scale represents the number of substitutions per site.

On the basis of the observed lack of concordance between the topology of the *HA* and *NA* gene phylogenies, we performed an analysis of the *NA* gene similar to that of Lewis et al. [7]. *NA* phylogenetic analysis revealed that all 16 Canadian *N2* genes were derived from a 2002 human origin N2 lineage (Figure 4). Overall, nucleotide sequence identity between the *NA* genes of all 16 Canadian viruses analyzed in this study ranged from 92.8% to 99.9% identity. Moreover, the *NA* gene of all 16 Canadian viruses showed an identity of 94.2% to 98.4% with Ontario swine viruses isolated between 2011 and 2012. When compared to the original Cluster IV trH3N2 (A/sw/ON/33853/05/H3N2) virus, the *NA* gene of 16 Canadian viruses showed a modest identity ranging from 94.1% to 97.5%.

The percentages of genetic relatedness of the 16 Canadian viruses to other published influenza viruses in the NCBI database are presented in Table S2. Further, we investigated the genetic basis for anticipated antigenic differences among 13 (H3) Canadian viruses. Therefore, we aligned aa sequences of the HA1 domain of 13 H3N2 viruses. Typically, antigenic differences are attributed to one or two aa changes located at positions (145, 155, 156, 158, 159, and 189) within the HA1 domain in antigenic sites A and B [22]. Here, we observed that three viruses (A/SW/MB/G1/2014, A/SW/AB/G9/2014, A/SW/ON/G15/2014) had a substitution at position 145 (N145K) that has been reported in human viruses [22]. Substitution (Y159N) has been observed in the (A/SW/ON/G15/2014) virus. The R189K substitution that plays an important role in the antigenicity of swine and human H3N2 viruses has been determined in three Canadian viruses (A/SW/MB/G1/2014, A/SW/ON/G10/2014, and A/SW/ON/G15/2014).

### 3.2. Amino Acid Variations in Antigenic Sites of the HA1 Region of 13 H3N2 Viruses

**Figure 5:**
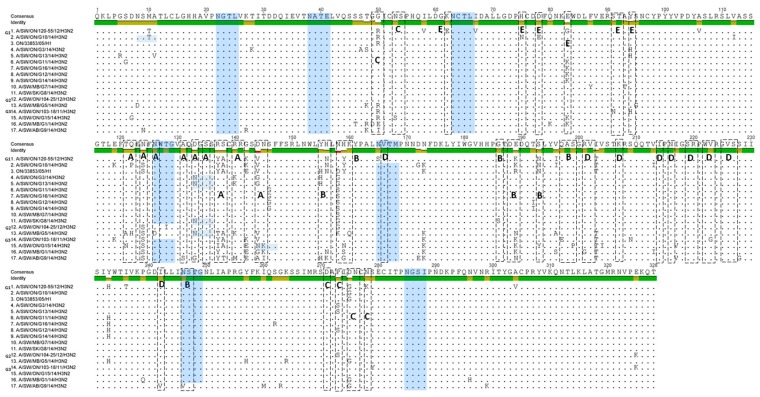
Alignment of the 13 Canadian H3 HA1 aa sequences without the signal peptide. Amino acids of the HA1 subunit of the 13 Canadian H3N2 viruses, prototype Cluster IV triple-reassortant H3N2 (trH3N2) virus (A/sw/ON/33853/05) and representatives of each group (G1, G2, and G3) of Ontario viruses isolated between 2011 and 2012 (G1-A/SW/ON/120-55/12), (G2-A/SW/ON/104-25/12), and (G3-A/SW/ON/103-18/11). Residues shown in boxes represent antigenic sites A, B, C, D, and E. Potential N-glycosylation sites are highlighted blue.

HA1 aa sequence alignment and variability in the antigenic epitopes of 13 Canadian H3N2 isolates are represented in Figure 5. Major aa changes have been observed in previously identified antigenic sites A, B, C, D, and E. We aligned the aa sequences of the 13 Canadian H3N2 viruses and compared them with the prototype cluster IV H3N2 virus (A/SW/ON/33853/05/H3N2).

**Figure 6:**
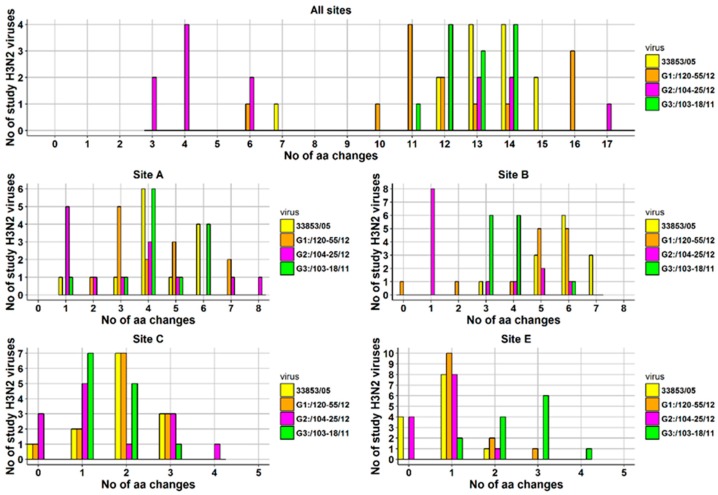
Number of amino-acid changes on all antigenic sites and on each antigenic site detected in 13 Canadian H3N2 IAVs isolated from clinical outbreaks in swine during 2014. Four viruses served as a basis for comparison: A/SW/ON/33853/05/H3N2 (**33853/05**), A/SW/ON/120-55/12/H3N2 (**G1:/120-55/12**), A/SW/ON/104-25/12/H3N2 (G2:/104-25/12), A/SW/ON/103-18/11/H3N2 (**G3:/103-18/11**). Note that antigenic site D has not been displayed as the total number of changes did not exceed two (max = 1) for comparison to all viruses except for **G3:/103-18/11**, where the total number of aa changes was 15 with a minimum of one and a maximum of two aa changes.

At the level of individual viruses, the number of aa changes varied between a minimum of seven and a maximum of 14 (Figure 6). The number of aa changes relative to the prototype cluster IV H3N2 virus for each H3N2 study virus and for each antigenic site is displayed as a histogram (Figure 6). Briefly, one virus was detected with seven aa changes on all antigenic sites, two with 12 aa changes, four with 13 and with 14 aa changes, and one with 15 aa changes. Most aa changes were detected in antigenic site A where 12/13 viruses had ≥3 aa changes (maximum = 6 aa changes), and antigenic site B where 13/13 viruses had ≥3 aa changes (maximum = 7 aa changes). The aa sequences of the viruses described in the current study were also compared to those of the three most recent Ontario H3N2 viruses isolated in 2011 and 2012, (A/SW/ON/103-18/11/H3N2, A/SW/ON/104-25/12/H3N2, and A/SW/ON/120-55/12/H3N2), provisionally named G3, G2, and G1, respectively. A substantial number of aa substitutions have been observed in previously identified antigenic sites and substitutions are presented in Figure 5 and Table S1. The most changes, were observed when 13 Canadian H3N2 viruses were compared with an Ontario virus from 2011, (A/SW/ON/103-18/11/H3N2) [15]. The number of aa changes relative to the latter virus for each antigenic site is also displayed in Figure 6.

Similar to the comparison with the referent Ontario strain from 2005, most aa changes were detected in antigenic site A where 12/13 viruses had ≥3 aa changes (maximum = 6 aa changes), and antigenic site B where 13/13 viruses had ≥3 aa changes (maximum = 6 aa changes). The HA1 aa sequence identity between the 13 H3N2 viruses and A/SW/ON/103-18/11/H3N2 ranged from 90.2% to 93.9%. The least changes in aa were observed when the study Canadian H3N2 viruses were compared with A/SW/ON/104-25/12/H3N2. Amino acid sequence identity of study viruses ranged from 90.5% to 98.2% when compared to A/SW/ON/104-25/12/H3N2. Most aa changes were still detected in antigenic sites A and B, where 7/13 viruses and 5/13 viruses had ≥3 aa changes, respectively. The lowest aa sequence identity (88.4% to 92.4%) was observed when HA1 of 13 Canadian H3N2 viruses was compared with HA1 of A/SW/ON/120-55/12/H3N2. Similar to the comparison with the referent Ontario strain from 2005, most aa changes were detected in antigenic site A where 12/13 viruses had ≥3 aa changes (maximum = 7 aa changes) and antigenic site B where 11/13 viruses had ≥3 aa changes (maximum = 6 aa changes). The number of aa changes per antigenic site and *in toto* when the 13 Canadian H3N2 viruses where compared to the four relevant Ontario strains is provided as a histogram (Figure 6).

The Canadian H3N2 viruses isolated in 2014 also exhibited various changes at antigenic site B. Amino acids at position 155–160 (HNLDYK) related to the prototype cluster IV virus were changed into eight different combinations, presented in Table S3. The N-glycosylation sites defined by the motif N-X-T/S, where X is not a Proline, have also been determined. The prototype cluster IV virus carries seven glycosylation sites (at 22, 38, 63, 126, 165, 246, and 285) highlighted in Figure 5. Seven potential N-glycosylation sites have also been predicted for seven Canadian H3N2 viruses (at 22, 38, 63, 126, 165, 246, and 285). Among these seven viruses, one (A/MB/ON/G5/14/H3N2) virus had a potential N-glycosylation site at residue 133, while the potential N-glycosylation site at residue 126 was missing. Additional N-glycosylation sites have been detected in a total of five Canadian viruses. Three viruses have an extra predicted N-glycosylation site at residue 133 (A/SW/ON/G3/14/H3N2, A/SW/SK/G8/14/H3N2, and A/SW/ON/G13/14/H3N2), one at residue 8 (A/SW/ON/G10/14/H3N2), and one at residue 144 (A/SW/ON/G15/14/H3N2). Six N-glycosylation sites have been predicted for one Canadian H3N2 virus A/SW/AB/G9/14/H3N2 (Figure 5). 

### 3.3. Amino Acid Variations in Antigenic Sites of the HA1 Region of Three H1N2 Viruses

**Figure 7:**
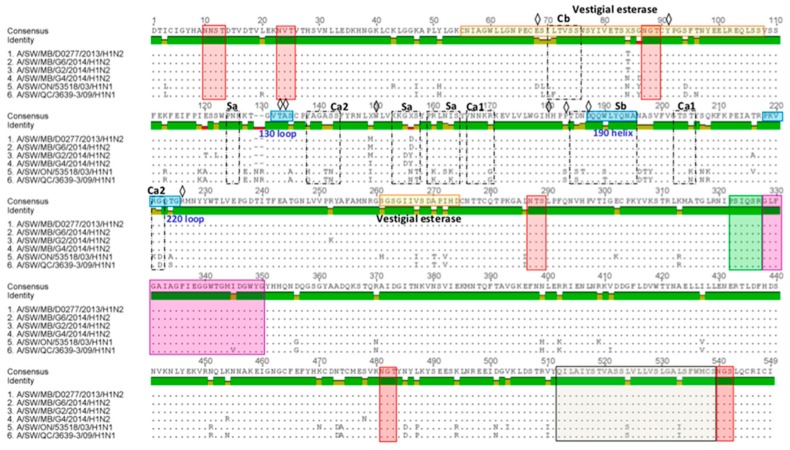
Predicted HA1 proteins of three Canadian H1 viruses isolated in 2014. The three Canadian H1 predicted HA1 proteins were aligned and compared to that of two Canadian viruses that belong to the αH1 cluster (A/sw/ON/53518/03 and A/sw/QC/3639/09). Additional comparison has been performed with the A/sw/MB/D0277/13 virus, with which they shared the highest aa sequence identity of 99%. Red boxes represent conserved gycosylation sites. The cleavage site and fusion peptide are shown in green and purple boxes, respectively. The residues 512 thorugh 539 (gray box) are the transmembrane region. Receptor-binding pocket residues are indicated by a diamond.

HA1 aa sequence alignment and variability in the antigenic epitopes of three Canadian H1N2 isolates are represented in Figure 7. Major aa changes have been observed at five known antigenic sites, Ca1, Ca2, Cb, Sa, and Sb. Amino acid sequences of three H1 predicted HA1 proteins were compared to those of two Canadian viruses that belong to the αH1 cluster (A/sw/ON/53518/03/H1N1) and (A/sw/QC/3639-3/09/H1N1). Amino acid sequences at the major antigenic sites of three Canadian H1N2 isolates were additionally compared to a (A/SW/MB/D0277/2013/H1N2) virus, with which they shared the highest aa sequence identity of 99%.

Each of the three Canadian H1N2 isolates had 16 aa changes within five antigenic sites, Ca1 (2), Ca2 (5), Cb (1), Sa (6), and Sb (2) when compared to the A/sw/ON/53518/03/H1N1. Canadian H1N2 isolates had 15 aa changes within five antigenic sites, Ca1 (2), Ca2 (4), Cb (2), Sa (5), and Sb (2) when compared to the A/sw/QC/3639-3/09/H1N1 virus. Only two aa changes at positions 155 and 156 at antigenic site Sa have been observed when HA1 of three Canadian H1N2 isolates were compared to those of the A/SW/MB/D0277/13/H1N2 virus. Further analysis of the receptor-binding pocket revealed the presence of 187D (or N), suggesting a “human” Neu5Acα2-6Gal receptor binding preference [23,24].

### 3.4. Amino Acids and Phylogenetic Analysis of the PB1-F2 Protein

**Figure 8:**
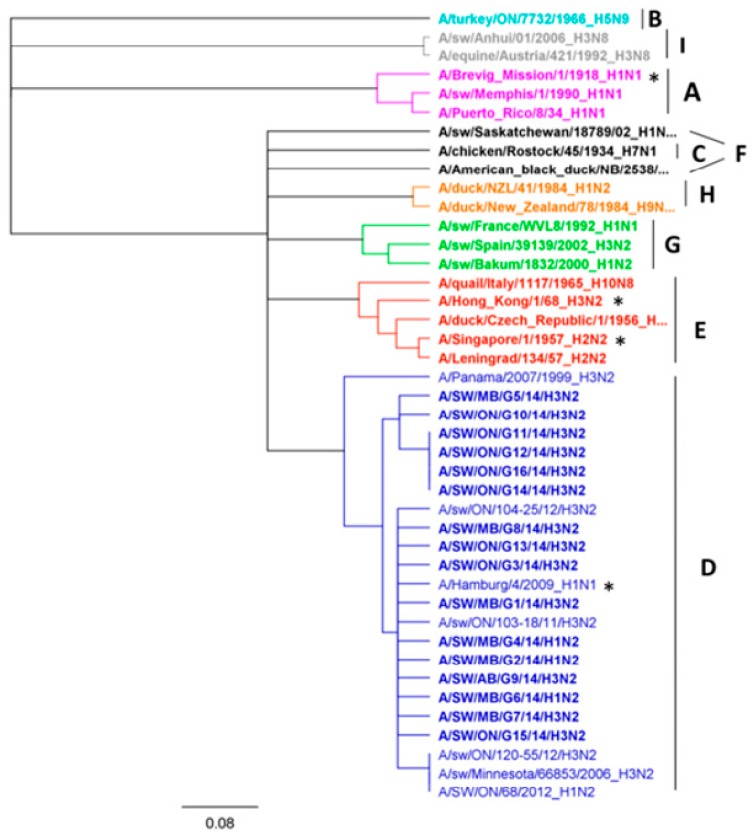
Phylogenetic analysis of 16 Canadian PB1 aa sequences. Representatives of the nine PB1 lineages ranging from A to I are shown. The pandemic strains of 1918 (H1N1), 1957 (H2N2), 1968 (H3N2) and 2009 (H1N1) are marked with asterisk. The scale represents the number of substitutions per site.

PB1-F2, an IAV protein encoded in the +1 alternate open reading frame (ORF) of the RNA polymerase subunit (*PB1* gene, segment 2), can be expressed as several different polypeptide lengths with a full size of mainly 87–101 aa [25]. There are several exceptions to this rule, such as the classical swine H1N1 virus, which comprises a truncated version of 11 aa [26], all human H1N1 viruses isolated after 1947 (57 aa) [27], all A(H1N1)pdm09 viruses (11 aa) [28], some human H3N2 viruses, and some North American swine triple reassortants [29], as well as some clades of avian IAV, for example, North American avian H5N2, H6N4, H6N6, and H13N2 viruses that belong to PB1-lineage F, and H13N6 viruses that represent PB1-lineage G [30]. Eight Canadian viruses analyzed in this study (seven H3N2 and one H1N2) covered the full-length of the 90 residues of PB1-F2. Three Canadian H3N2 viruses had a PB1-F2 of 57 residues and one H3N2 virus had 34 residues. Four viruses, two H3N2 and two H1N2 viruses, harbored a truncated form of PB1-F2 containing eight aa, lacking the mitochondrial targeting sequence (MTS) located in the C-terminal region. More detailed protein sequence analysis of all 16 Canadian PB1-F2 proteins exposed the absence of the N66S mutation, reported in the highly pathogenic H5N1 and 1918 Spanish flu H1N1 viruses and associated with increased pathogenicity in mice. Additional aa changes associated with lower pathogenicity in avian IAVs, such as T51M, V56A, and E87G, have also been investigated. Eleven Canadian viruses have a T51M substitution, while six have an E87G substitution. The V56 and E87 substitutions have been determined in 11 and two Canadian viruses, respectively. Phylogenetic analysis of all 16 Canadian PB1-F2 aa sequences showed that these viruses belong to lineage D (Figure 8).

### 3.5. Resistance-Associated Mutations

**Figure 9:**
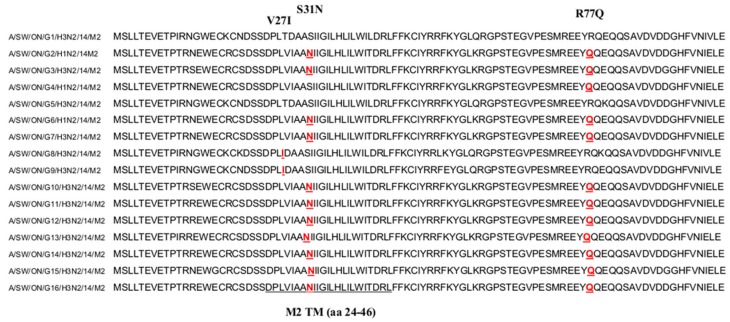
Alignment analysis of M2 sequences of 16 IAVs from swine isolated in Canada showing aa substitutions V27I, S31N, inside the transmembrane domain of the M2 protein, and the R77Q aa substitution.

Currently, two classes of influenza antivirals have been approved in many countries around the globe: the adamantanes and the neuraminidase inhibitors (NAIs). In order to determine resistance-associated mutations to these two classes of drugs, we aligned and analyzed the aa sequences of the M and NA proteins of all 16 Canadian isolates. Commonly, resistance to adamantanes is associated with seven aa residues in the M2 protein (L26F, V27A, A30T/V, S31N, G34E, L38F, and R77Q) [31,32]. Sequence alignment analysis of all 16 Canadian isolates has shown that 11 of 16 isolates do have an S31N mutation in the M2 protein, which is most frequently associated with adamantine resistance. Additional mutations, such as R77Q and V27I, have been detected in 12 and two isolates, respectively. The residues conferring resistance to adamantanes are shown in Figure 9. The NA mutations, such as E119V, R292K, and N294S, associated with oseltamivir resistance in the N2 subtype, were not detected in the NA protein of any of the 16 Canadian isolates.

## 4. Discussion

The influenza virus continues to be one of the most important viral pathogens in Canadian swine production and also potentially poses a risk to other host species, including humans. The HA protein represents the primary target of protective immune responses and is the major component in swine influenza A vaccine. Molecular characterization of swine influenza A isolates and their cross-reactivity is an important step in order to examine the genetic basis for antigenic differences among circulating swine influenza A isolates and identify aa substitutions that may lead to immune escape and vaccine failure in pigs. Continuous identification of new clusters/clades among H3 and H1 viruses demonstrates the need for such research to capture the full diversity of IAVs in swine in Canada and the importance of antigenic drift in the diversity and emergence of new antigenic variants in swine, which complicates vaccine design. Here, we describe the genetic and predicted antigenic characterization of 13 Canadian H3N2 and three Canadian H1N2 viruses isolated from swine in 2014. Characterization of the 13 Canadian H3N2 viruses clearly indicates reassortment of gene segments between the North American swine trH3N2 from Cluster IV and the A(H1N1)pdm09. We detected five unique groups of H3N2 reassortants, based on H3N2/H1N1pdm(09) gene combinations. Structurally similar viruses have already been reported on various occasions since 2009 [33,34,35]. Within cluster IV, eight H3N2 viruses belong to the IV-C cluster, one H3N2 virus belongs to the IV-B cluster, and one to the IV-D cluster. Interestingly, three H3N2 viruses could not be grouped to any known cluster IV (A to F) based on standard sequences used in this study. The latter three viruses seemed to be distantly related to the prototype cluster IV trH3N2 virus (A/sw/ON/33853/05). The search of Genbank revealed that at least some of the three viruses are very similar to IAV isolates detected recently in other North American swine populations and they (particularly A/SW/ON/G15/2014) cannot be considered as novel strains. Phylogenetic analysis of the *HA* gene of three H1N2 viruses isolated in 2014 from Manitoba revealed that they were within the H1α cluster. These three H1N2 alpha viruses contained two aa deletions at positions 129 and 130, according to the H1 numbering system. This finding is in agreement with report of Redies et al. [13] where these unique deletions have been observed as dominant among swine H1N2 IAV in Western Canada.

Detailed HA1 antigenic site analysis of 13 H3N2 viruses has been performed employing the three Ontario H3N2 viruses, representatives of each group (G1, G2, and G3) isolated in 2011 and 2012 [15]. A significant number of aa substitutions have been recorded in previously identified antigenic sites, confirming the mercurial nature of influenza viruses, which undergo continuous antigenic evolution. Additionally, the percentage of aa identity of HA1 between the prototype cluster IV trH3N2 virus (A/sw/ON/33853/05) and 13 Canadian H3N2 viruses ranged from 93% to 96%. The HA1 aa sequences of all 13 H3N2 viruses, when compared to the prototype cluster IV virus, showed a minimum of seven and maximum of 15 aa changes at five antigenic sites, A to E. Importantly, one to three aa changes in the HA1 of H1N1 or H3N2 virus are sufficient to reduce the cross reactivity and efficacy of inactivated vaccine [36,37]. Similar findings were observed when H3N2 field viruses were compared to previous Canadian isolates grouped into G1 and G3 groups [15]. This further suggests that exposure to some contemporary or recently identified strains would not necessarily result in effective protection against strains detected in this study. Further experimental and field studies investigating within-herd dynamics would be useful to understand possible co-circulation of different influenza viruses. This co-circulation was recorded soon after emergence of H3N2 in North American swine populations [38] and has been observed on numerous occasions since then. The least number of changes was observed when field viruses were compared to the G2 representative. This finding in itself is not surprising since a substantial proportion of the current study viruses were classified into cluster IV-C, together with the isolate that is representative of Group 2. Further studies and more intense surveillance should be conducted to elucidate whether indeed the majority of current H3N2 field isolates could be grouped into the cluster IV-C, or whether this was a reflection of possible selection bias of the current study population. Although it would be tempting to suggest that a candidate from cluster IV-C would be suitable for regional vaccine production, some caution is needed even on the basis of this study population alone. The minimum number of all aa changes when field viruses were compared to the G2 (cluster IV-C) representative was three, which suggests that these viruses are indeed subject to ongoing drift and diversification even within the subcluster. Thus, selecting candidates for possible regional vaccine based on subcluster membership alone might not yield perfect match with circulating strains, and is perhaps not realistic under current conditions. 

The HA1 antigenic site analysis of three H1N2 viruses was performed employing the virus A/SW/MB/D0277/13/H1N1, with which they shared the highest aa sequence identity of 99%. Comparison revealed two aa changes at antigenic site Sa, aa positions 155 and 156. Three H1N2 viruses from this study were also isolated in Manitoba, therefore such a high percentage of identity was not surprising, although changes in antigenic epitope had already started to appear in these viruses.

However, IAVs commonly escape antibody-mediated neutralization by gathering mutations in HA, a process known as antigenic drift. The research of Lewis et al. [7] showed that substitutions which resulted in marked antigenic differences were attributed in most cases to one or two aa changes in the HA-1 domain, located at six aa positions (145, 155, 156, 158, 159, and 189). This finding was remarkably similar to the seven key aa changes recently identified in human antigenic switches from 1968 to 2003: 145, 155, 156, 158, 159, 189, and 193 [22]. Three viruses from this study (A/SW/MB/G1/2014, A/SW/AB/G9/2014, A/SW/ON/G15/2014) had a substitution at position 145 (N145K), responsible for major antigenic changes in human viruses [22]. Further analysis of the Canadian 2014 viruses showed the presence of an R189K substitution within three viruses (A/SW/MB/G1/2014, A/SW/ON/G10/2014, and A/SW/ON/G15/2014). It has been reported that position 189 defines the antigenic difference between the H3-IV (B) and H3-IV (C) swine antigenic clusters [7]. The role of the R189K substitution has been explored further by Ye et al. [39], confirming that the R189K mutation plays a vital role in the antigenicity of swine and human H3N2 IAVs. Identification of this antigenic determinant could help us rapidly identify antigenic variants in influenza surveillance. The antigenic cartography of Ye et al. [39] demonstrated that the R189K mutation in the HA of H3N2 IAV contributed to the antigenic drift separating viruses within two clusters. The 189 aa position has been consistently identified as cluster-defining in other species, as well. According to Koel et al. [22], although position 189 seems to be more consistently identified among different species and in different studies, it is clear that it is not the sole position responsible for cluster transition substitutions in human and swine H3N2 viruses. The aa changes might cause structural differences in the HA, leading to receptor-binding restrictions in different hosts, differences in adaptive immune recognition, or a combination of both. Since recent publications of Anderson et al. [6] and Lewis et al. [7] have shown that as few as one or two aa changes in the HA-1 domain are enough to drive a more than 4-fold reduction in relative cross-reactivity and result in vaccine failure, it is important to continue to systematically monitor the evolution of swine IAVs for vaccine strain updates. The *N*-glycosylation sites on the HA protein could also contribute to antibody escape by binding oligosaccharides and consequently masking the antigenic sites to antibodies, although the exact mechanism by which this is achieved is an area of active research [40,41]. Predicted *N*-glycosylation sites for H3N2 viruses have been in general agreement with previously reported predicted sites for Canadian H3N2 viruses isolated from turkeys [42]. However, several study viruses were predicted to have either lost, or gained *N*-glycosylation sites. Therefore, current results suggest that the 2014 Canadian H3N2 viruses could show some variability in the position of predicted *N*-glycosylation sites with possible implications on the interaction between the host’s immune response and virus. This, therefore, needs to be monitored and evaluated using a more comprehensive study with a larger viral population and over a longer period of time.

Although continuing evolution is most prominent in the surface glycoproteins, it has also been reported in each of the eight segments of the influenza viruses. One of them is the PB1-F2 protein, in which translation starts from the fourth initiation codon, surpassing three other initiation codons of the *PB1* gene [43]. An intriguing genomic feature is the varying lengths of the PB1-F2 protein. Whereas highly pathogenic strains, such as the H5N1 subtype, express a longer polypeptide of 90 aa, the low pathogenic subtypes tend to express a C-terminal truncated version encoding 57 aa [25]. The 1918 H1N1 contained a complete *PB1*-F2 protein, while most H1N1 viruses have an incomplete PB1-F2 protein with truncation either at the N- or C- terminal end [44,45]. Interestingly, the N-terminal end of the PB1-F2 is preserved in human hosts, while the C-terminal end is preserved in swine [46]. Varying length has also been observed among Canadian viruses. Eight of 16 Canadian viruses exhibited a 90-aa long PB1-F2 protein (seven H3N2 and one H1N2), while three (H3N2) viruses had 57 aa, one (H3N2) virus had 34 aa, and four (two H3N2 and two H1N1) viruses had an 8-aa long PB1-F2 protein. According to Yoshizumi et al. [25], the full-length version of PB1-F2 translocates into mitochondria, and this translocation is strongly correlated with impaired innate cellular immunity. PB1-F2 variants lacking a C-terminal polypeptide, which are frequently found in low pathogenic subtypes, do not affect mitochondrial function [25]. An N66S (arginine to serine) substitution in the C-terminal region of the protein associated with apoptotic function of the protein has been absent in all 16 Canadian viruses.

Amantadine (Symmetrel) and rimantadine (Flumadine) were the first influenza antivirals approved for the treatment and prophylaxis of influenza [47]. The amantadine and its derivate rimantadine inhibit viral replication by blocking the activity of the M2 ion channel that is essential for viral uncoating following entry into the cell [48]. These drugs are not effective against influenza B viruses because of structural differences between influenza A and B viruses [48]. One of the most common adamantadine resistance mutations is S31N in human, avian, and swine IAVs [49]. About 95% of adamantadine-resistant influenza viruses contain S31N aa mutations, while only 1% have V27A in the M2 protein. Drug resistance mutations related to other aa positions (L26F, A30T, G34E, and L38F) are particularly rare (<0.2%) [49]. In agreement with this finding, 11 of 16 Canadian IAVs exhibited the presence of an S31N mutation. Previously reported, a non-synonymous mutation V271 [50,51], that retains drug resistant properties, has also been detected in the *M2* gene of two Canadian IAVs. The R77Q mutation reported by Krumbholz et al. [52] was detected in M2 sequences of European swine IAVs. Their analysis supported the finding that S31N and R77Q are conditionally dependent at a significant level. Interestingly, the R77Q mutation was detected in 12 of the 16 Canadian IAVs, and only in one case was not accompanied by the S31N mutation. On the basis of our analysis, we can conclude that at least 11 Canadian viruses of the 16 evaluated can be expected to offer resistance to amantadine and rimantadine.

The information presented in this study could be of interest to veterinary practitioners. On the basis of a limited number of samples obtained from clinical outbreaks, only isolates from western Canada (Alberta, Manitoba, and Saskatchewan) had all gene segments most similar to the trH3N2 isolates. This is likely due to the nature of the study population since a recent study from Ontario indicated that H3N2 strains with all gene segments associated with trH3N2 virus were present [15]. The H1N2 virus has been detected in Manitoba which is not surprising due to recent reports suggesting the spread of this particular variant of H1N2 in western Canada [13]. The gene combination representing a distinct combination of gene segments did not appear as a good predictor of expected antigenic grouping. For example, the four viruses that are characterized as having all segments originating from trH3N2 viruses were placed in four different subclusters based on the aa sequence of their HA1 region. Some of the newly detected recombinants were indeed all classified to the same expected antigenic cluster (such as a large group of Ontario strains in cluster IV-C), however, other types of recombinant viruses were also grouped in the same predicted antigenic subcluster. This reinforces the idea that for good clinical decision making, thorough understanding of all circulating strains in a given production system or a geographical area is needed, and their detailed analysis would be beneficial. Further research is also needed with respect to isolates that gained or lost predicted N-glycosylation sites. Interestingly, viruses that were predicted to have gained N-glycosylation sites were either grouped into the cluster IV-C of the H3N2 subtype, or represented variants that could not be classified into any of the existing H3N2 subclusters.

This study has several limitations. When evaluating the proportion of subtypes in the study, it needs to be emphasized that they may not represent a proportional distribution of subtypes in the source population, and the lack of H1N1 viruses was particularly obvious in this respect. This could be due to several reasons. First, the viruses characterized in this study are aimed for inclusion into autogenous vaccines and represent viruses from the subset of herds where influenza was considered of sufficient importance for such an infection control measure. Hence, they represent a biased section of the viral and swine population. Thus, observed frequencies could be due to the selection bias that occurred at different stages. Second, this proportional distribution might be representative of the influenza situation at the time the study was conducted, and of the emerging viruses that caused clinical problems in the source herds. An example would be H1N2 that was reported to spread in parts of population [13]. Third, with a small study, it is not unusual to observe deviations from expected values based on population averages. Another set of limitations is a result of the type of study material. The viruses considered for this study were originally isolated with the ultimate purpose to serve in the production of the autogenous vaccines. In the case that originally submitted material contained multiple viruses, the possibility that a virus with the highest affinity for the cell culture was preferentially isolated cannot be excluded. The propagation in cell culture could have also introduced certain mutations. Nonetheless, the purpose of the work was to describe viruses that were causing sufficient clinical issues to prompt practitioners and herd owners to produce the autogenous vaccines, and the source of the study material was aligned with this objective.

In conclusion, the results of this study showed that at least five unique groups of H3N2 reassortants were based on H3N2/H1N1pdm(09) gene combination. All 13 H3N2 viruses belonged to cluster IV, eight H3N2 viruses were part of the IV-C cluster, one virus belonged to the IV-B cluster, and one virus to the IV-D cluster. Three H3N2 viruses could not be clearly classified into any known cluster IV (A to F) based on standards used in this study, although they appeared to be most related to the original cluster IV viruses. However, for at least one of them, very similar viruses have already been detected in other parts of North American swine populations, suggesting that they may not be novel viruses. Three H1N2 viruses belonged to the H1α cluster and contained two aa deletions at aa positions 129 and 130. On the basis of aa sequence analysis of the M2 protein, the majority of Canadian H3N2 and H1N2 viruses (at least 69%) were expected to offer resistance to adamantadine derivatives. On the basis of aa sequence analysis of the NA protein, none of the study viruses were expected to be resistant to oseltamivir.

## Figures and Tables

**Table 1:** Influenza A viruses (IAVs) isolated from swine in Alberta, Manitoba, Ontario, and Saskatchewan in 2014.

Isolate	Subtype
A/SW/MB/G1/2014	H3N2
A/SW/MB/G2/2014	H1N2
A/SW/ON/G3/2014	H3N2
A/SW/MB/G4/2014	H1N2
A/SW/MB/G5/2014	H3N2
A/SW/MB/G6/2014	H1N2
A/SW/MB/G7/2014	H3N2
A/SW/SK/G8/2014	H3N2
A/SW/AB/G9/2014	H3N2
A/SW/ON/G10/2014	H3N2
A/SW/ON/G11/2014	H3N2
A/SW/ON/G12/2014	H3N2
A/SW/ON/G13/2014	H3N2
A/SW/ON/G14/2014	H3N2
A/SW/ON/G15/2014	H3N2
A/SW/ON/G16/2014	H3N2

**Table 2:** Genome constellations identified in 13 Canadian H3N2 and three H1N2 viruses isolated from clinical outbreaks in swine in Alberta, Manitoba, Ontario and Saskatchewan in 2014.

Virus *	*HA*	*NA*	*PA*	*PB1*	*PB2*	*NP*	*M*	*NS*
/SW/MB/G1; /SW/MB/G5, /SW/SK/G8, /SW/AB/G9								
/SW/MB/G2, /SW/MB/G4, /SW/MB/G6	αH1							
/SW/ON/G3								
/SW/MB/G7, /SW/ON/G15								
/SW/ON/G10								
/SW/ON/G11; /SW/ON/G12; /SW/ON/G13; /SW/ON/G14; /SW/ON/G16								

Green = triple reassortant H3N2 (trH3N2); Red = pandemic H1N1; Blue = αH1; * names of viruses have been shortened due to space limitation

## Supplementary Materials

The following are available online at www.mdpi.com/1999-4915/9/3/55/s1, Table S1: Amino acid sequence substitutions observed in the HA1 of 13 H3N2 viruses isolated from Canadian swine; Table S2: Top BLAST matches of the eight gene segments from 13 Canadian H3N2 and three H1N2 viruses obtained from NCBI influenza A virus nucleotides and amino acid database; Table S3: Unique changes exhibited at antigenic site B at amino acid position 155–160, compared to prototype cluster IV virus; Figure S4: The H3N2 HA phylogeny of Cluster IV from A to F (indicated on right), based on amino acid (aa) sequences of the HA1 region; Figure S5: The H3N2 HA phylogeny of Cluster IV from A to F (indicated on right), based on amino acid (aa) sequences of the HA1 region; Figure S6: Phylogenetic analysis of the full hemagglutinin (HA) gene of three Canadian H1N2 viruses isolated from swine in 2014 (black) based on nucleotide sequences; Figure S7: Phylogenetic analysis of the full hemagglutinin (*HA*) gene of three Canadian H1N2 viruses isolated from swine in 2014 (black) based on nucleotide sequences.
