# Supplementary material for: Virological Surveillance of Influenza A Subtypes Isolated in 2014 from Clinical Outbreaks in Canadian Swine
Source: Viruses. 2017 Mar 21;9(3):55. doi: 10.3390/v9030055 (PMC5371810; doi:10.3390/v9030055)
Supplement: Supplementary file 1 [file viruses-09-00055-s001.pdf]

Table S1. Top BLAST matches of the 8 gene segments from 13 Canadian H3N2 and 3 H1N2 viruses obtained from NCBI influenza A virus nucleotides and amino acid database.

		**A/SW/MB/G1/2014(H3N2)**	**A/SW/MB/G2/2014(H1N2)**	**A/SW/ON/G3/2014(H3N2)**
**gene**				
**PB2**	**NA**[a]	99% A/sw/MB/D0280/13(H3N2)	99% A/sw/MB/D0285/13(H1N2)	99% A/sw/MN/SG1238/05(H3N2
	**AA**[b]	100% A/sw/MB/D0280/13(H3N2)	100% A/sw/IA/13C038/13(H3N2)	99% A/turkey/OH/313053/04(H3N2)
**PB1**	**NA**	99% A/sw/MB/D0280/13(H3N2)	99% A/sw/MB/D0335/14(H1N2)	99% A/sw/MI/A01202035/11(H1N2)
	**AA**	99% A/sw/MB/D0280/13(H3N2)	99% /sw/MB/D0357/2014(H1N2)	99% A/sw/NC/SG1278/07(H1N1)
**PA**	**NA**	99% A/sw/MB/D0280/13(H3N2)	99% A/sw/MB/D0335/14(H1N2)	99% A/Bangkok/SIMI501/09(H1N1)
	**AA**	99% A/sw/MB/D0280/13(H3N2)	99% A/sw/MB/D0351/14(H1N2)	99% A/Singapore/DMS26/09(H1N1)
**HA**	**NA**	100% A/sw/MM/D0280/13(H3N2)	99% A/sw/MB/D0347/14(H1N2)	98% A/sw/IN/A01202866/11(H3N2)
	**AA**	100% A/sw/MB/D0280/13(H3N2)	99% A/sw/MB/D0347/14(H1N2)	98% A/sw/IN/A01202866/11(H3N2)
**NP**	**NA**	100% A/sw/MB/D0280/13(H3N2)	99% A/sw/MB/D0347/14(H1N2)	98% A/sw/ON/204-76/12(H3N2)
	**AA**	99% A/ON/1252/07(H3N2)	99% A/ON/1252/07(H3N2)	99% A/sw/Mie/R02/12(H1N2)
**NA**	**NA**	100% A/sw/MB/D0280/13(H3N2)	A/sw/MB/D0335/14(H1N2)	99% A/sw/QC/4001/05(H3N2)
	**AA**	100% A/sw/MB/D0280/13(H3N2)	99% A/sw/MB/D0335/14(H1N2)	99% A/sw/MB/00446/05(H3N2)
**M**	**NA**	100% A/sw/MB/D0280/13(H3N2)	100% A/sw/MB/D0335/14(H1N2)	99% A/Singapore/DMS56/09(H1N1)
**M1**	**AA**	100% A/ferret/IA/15828/08(H1N1)	100% A/CA/07/09(H1N1)	100% A/CA/07/09(H1N1)
**M2**	**AA**	100% A/sw/MB/D0089/12(H3N2)	100% A/NY/4761/09(H1N1)	100% A/sw/NC/A01049163/10(H1N1)
**NS**	**NA**	100% A/sw/MB/D0280/13(H3N2)	99% A/sw/MB/D0335/14(H1N2)	99% A/sw/IN/A01202866/11(H3N2)
	**AA**	100% A/sw/MB/D0280/13(H3N2)	100% A/sw/IA/13C019/13(H3N2)	99% A/sw/IN/A01202866/11(H3N2)
		**A/SW/MB/G4/2014\(H1N2)**	**A/SW/MB/G5/2014(H3N2)**	**A/SW/MB/G6/2014(H1N2)**
**gene**				
PB2	**NAa**	99% A/sw/MB/D0339/14(H1N2)	97% A/sw/MN/SG1238/05(H3N2)	99% A/sw/MB/D0328/14(H1N2)
	**AAb**	100% A/sw/IA/13C038/13(H3N2)	99% A/sw/MN/001444/07(H3N2)	100% A/sw/IA/13C038/13(H3N2)
PB1	**NA**	99% A/sw/MB/D0298/13(H1N2)	98% A/sw/MB/01781/07(H1N1)	100% A/sw/MB/D0285/13(H1N2)
	**AA**	99% A/sw/MB/D0268/13(H1N2)	99% A/sw/NC/SG1338/09(H3N2)	99% A/sw/MB/D0357/14(H1N2)
PA	**NA**	99% A/sw/MB/D0298/13(H1N2)	97% A/sw/MB/00446/05(H3N2)	99% A/sw/MB/D0277/13(H1N2)
	**AA**	100% A/sw/MB/D0267/13(H1N2)	99% A/sw/IA/H02NJ56371/02(H1N1)	100% A/sw/IA/13C038/13(H3N2)
HA	**NA**	99% A/sw/MB/D0339/14(H1N2)	96% A/sw/IA/SG1469/04(H3N2)	99% A/sw/MB/D0296/13(H1N2)
	**AA**	99% A/sw/MB/D0298/13(H1N2)	96% A/sw/NC/SG1230/05(H3N2)	99% A/sw/MB/D0277/2013(H1N2)
NP	**NA**	100% A/sw/MB/D0295/13(H1N2)	97% A/sw/AB/SG1405/05(H3N2)	99% A/sw/MB/D0293/2013(H1N2
	**AA**	99% A/ON/1252/07(H3N2)	99% A/ON/1252/07(H3N2)	99% A/ON/1252/07(H3N2)
NA	**NA**	99% A/sw/MB/D0295/13(H1N2)	97% A/sw/NC/SG1464/04(H3N2)	100% A/sw/MB/D0285/13(H1N2)
	**AA**	100% A/sw/MB/D0273/13(H1N2)	97% A/turkey/IL/04(H3N2)	100% A/sw/MB/D0285/13(H1N2)
M	**NA**	100% A/sw/MB/D0298/13(H1N2)	98% A/sw/KY/SG1118/03(mixed)	99% A/sw/MB/D0335/14(H1N2)
M1	**AA**	100% A/CA/07/09(H1N1)	100% A/sw/IA/A01049031/10(H3N2)	100% A/CA/07/09(H1N1)
M2	**AA**	100% A/sw/MB/D0267/13(H1N2)	100% A/sw/IA/A01049317/10(H3N2)	100% A/NY/4761/09(H1N1)
NS	**NA**	99% A/sw/MB/D0339/2014(H1N2)	99% A/sw/MN/SG1236/05(H3N2)	100% A/sw/MB/D0335/14(H1N2)
	**AA**	100% A/sw/MB/D0267/13(H1N2)	99% A/ON/RV1273/05(H3N2)	100% A/sw/IA/13C019/13(H3N2)

		**A/SW/MB/G7/2014(H3N2)**	**A/SW/SK/G8/2014(H3N2)**	**A/SW/AB/G9/2014(H3N2)**
**gene**				
PB2	**NAa**	99% A/sw/MB/D0285/13(H1N2)	99% A/sw/SK/SD0051/14(H3N2)	97% A/sw/MN/SG1238/05(H3N2)
	**AAb**	99% A/sw/IA/13C038/13(H3N2)	100% A/sw/SK/SD0051/14(H3N2)	99% A/sw/NC/SG1232/05(H1N2)
PB1	**NA**	99% A/sw/MB/D0285/13(H1N2)	99% A/sw/SK/SD0059/14(H3N2)	98% A/sw/QC/00686/05(H3N2)
	**AA**	99% A/sw/MB/D0368/13(H3N2)	99% A/sw/SK/SD0059/14(H3N2)	99% A/sw/IN/A00968359/12(H3N2)
PA	**NA**	99% A/sw/MB/D0277/13(H1N2)	99% A/sw/SK/SD0025/14(H3N2)	97% A/sw/KS/12-165022/12(H3N2)
	**AA**	100% A/sw/IA/13C038/13(H3N2)	99% A/sw/SK/SD0025/14(H3N2)	98% A/sw/KS/12-165022/12(H3N2)
HA	**NA**	99% A/sw/ON/105-56/12(H3N2)	99% A/sw/ON/105-56/12(H3N2)	96% A/sw/MN/A01201894/11(H3N2)
	**AA**	99% A/sw/ON/104-25/12(H3N2)	99% A/sw/ON/105-56/12(H3N2)	96% A/sw/KS/12-165022/12(H3N2)
NP	**NA**	99% A/sw/MB/D0348/2014(H1N2)	99% A/sw/SK/SD0051/14(H3N2)	98% A/sw/MB/00446/05(H3N2)
	**AA**	99% A/ON/1252/07(H3N2)	99% A/ON/1252/07(H3N2)	99% A/ON/1252/07(H3N2)
NA	**NA**	99% A/sw/MB/D0287/13(mixed)	99% A/swine/QC/1257774/10(H3N2)	97% A/sw/KS/11-110529/11(H3N2)
	**AA**	99% A/sw/MB/D0285/13(H1N2)	99% A/sw/QC/1257774/10(H3N2)	98% A/sw/MN/A01201894/11(H3N2)
M	**NA**	100% A/sw/MB/D0335/14(H1N2)	99% A/sw/SK/SD0059/14(H3N2)	98% A/sw/KS/12-165022/12(H3N2)
M1	**AA**	100% A/CA/07/09(H1N1)	100% A/ferret/IA/15828/08(H1N1)	98% A/sw/IL/100085A/01 (H1N2)
M2	**AA**	100% A/NY/4761/09(H1N1)	100% A/sw/SK/SD0012/13(H3N2)	100% A/sw/KS/12-165022/12(H3N2)
NS	**NA**	100% A/sw/MB/D0335/14(H1N2)	99% A/sw/SK/SD0025/14(H3N2)	97% A/sw/IA/A01202889/11(H3N2)
	**AA**	100% A/sw/IA/13C019/13(H3N2)	99% A/sw/SK/SD0007/12(mixed)	95% A/sw/IA/A01202889/11(H3N2)

		**A/SW/ON/G10/2014(H3N2)**	**A/SW/ON/G11/2014(H3N2)**	**A/SW/ON/G12/2014(H3N2)**
**gene**				
PB2	**NAa**	98% A/ON/RV1273/05(H3N2)	99% A/sw/ON/107-22/12(H3N2)	99% A/sw/ON/107-22/12(H3N2)
	**AAb**	99% A/sw/MB/02862/09(H3N2)	99% A/sw/ON/107-22/12(H3N2)	99% A/sw/ON/107-22/12(H3N2)
PB1	**NA**	98% A/sw/MB/00446/05(H3N2)	99% A/turkey/ON/FAV-10/11(H3N2)	99% A/turkey/ON/FAV-10/11(H3N2)
	**AA**	99% A/sw/MB/D0083/13(H3N2)	99% A/sw/ON/105-56/12(H3N2)	99% A/sw/ON/105-56/12(H3N2)
PA	**NA**	97% A/turkey/ON/31232/05(H3N2)	99% A/sw/ON/204-76/12(H3N2)	99% A/sw/ON/204-76/12(H3N2)
	**AA**	98% A/sw/MN/SG1144/06(H1N1)	99% A/sw/MI/A01202036/11(H1N1)	99% A/sw/MI/A01202036/11(H1N1)
HA	**NA**	97% A/sw/MN/SG1238/05(H3N2)	98% A/sw/ON/105-56/12(H3N2)	99% A/sw/ON/105-56/12(H3N2)
	**AA**	97% A/sw/MB/01782/07(H3N2)	98% A/sw/ON/105-56/12(H3N2)	98% A/sw/ON/105-56/12(H3N2)
NP	**NA**	99% A/ON/315181/09(H1N1)	A/sw/ON/204-76/12(H3N2)	99% A/sw/ON/204-76/12(H3N2)
	**AA**	99% A/sw/Yamagata/12/10(H1N1)	99% A/sw/AB/OTH-33-14/09(H1N1	99% A/sw/Mie/R02/12(H1N2)
NA	**NA**	97% A/sw/QC/00686/05(H3N2)	98% A/sw/ON/107-22/12(H3N2)	98% A/sw/ON/107-22/12(H3N2)
	**AA**	98% A/turkey/OH/313053/04(H3N2)	97% A/sw/ON/107-22/12(H3N2)	97% A/sw/ON/107-22/12(H3N2)
M	**NA**	99% A/sw/ON/46/12(H1N1)	99% A/sw/MI/A01104086/15(mixed)	99% A/sw/MI/A01104086/15(mixed)
M1	**AA**	100% A/CAD-ON/RV1527/09(H1N1)	100% A/sw/KS/A01377287/14(H1N2)	100% A/sw/KS/A01377287/14(H1N2)
M2	**AA**	100% A/CA/07/09(H1N1)	100% A/ON/3620/10(H1N1)	100% A/ON/3620/10(H1N1)
NS	**NA**	99% A/sw/ON/46/12(H1N1)	99% A/sw/ON/114-13/12(H3N2)	99% A/sw/ON/114-13/12(H3N2)
	**AA**	99% A/sw/ON/46/12(H1N1)	100% A/sw/ON/114-13/12(H3N2)	99% A/sw/ON/114-13/12(H3N2)

gene		**A/SW/ON/G13/2014**	**A/SW/ON/G14/2014**	**A/SW/ON/G15/2014**
PB2	**NAa**	99% A/sw/IN/A01202867/11(H3N2)	99% A/sw/ON/107-22/12(H3N2)	99% A/sw/MB/D0191/13(H3N2)
	**AAb**	99% A/sw/IN/A01260185/13(H3N2)	99% A/sw/ON/107-22/12(H3N2)	99% A/sw/IA/13C038/13(H3N2)
PB1	**NA**	99% A/sw/IN/A01260185/13(H3N2)	99% A/turkey/ON/FAV-10/11(H3N2)	99% A/sw/MB/D0201/13(H3N2)
	**AA**	99% A/sw/MB/D0379/15(H3N2)	99% A/sw/ON/105-56/12(H3N2)	99% A/sw/IA/13C038/13(H3N2)
PA	**NA**	99% A/sw/IN/A01202867/11(H3N2)	99% A/sw/ON/204-76/12(H3N2)	99% A/sw/MB/D0201/13(H3N2)
	**AA**	99% A/sw/MI/A01202036/11(H1N1)	99% A/sw/MI/A01202036/11(H1N1)	99% A/sw/IA/13C038/13(H3N2)
HA	**NA**	98% A/sw/IN/A01202866/11(H3N2)	99% A/sw/ON/105-56/2012(H3N2	99% A/sw/IA/A01472644/15(H3N2)
	**AA**	99% A/sw/IN/A01202866/11(H3N2)	98% A/sw/ON/104-25/12(H3N2)	99% A/sw/IN/A01812159/16(H3N2)
NP	**NA**	99% A/sw/ON/204-76/12(H3N2)	99% A/sw/ON/204-76/12(H3N2)	99% A/sw/MB/D0302/13(H1N2)
	**AA**	99% A/sw/AB/OTH-33-14/09(H1N1)	99% A/sw/AB/OTH-33-14/09(H1N1)	99% A/ON/1252/07(H3N2)
NA	**NA**	99% A/sw/IN/A01202867/11(H3N2)	98% A/sw/ON/107-22/12(H3N2)	99% A/sw/MB/D0218/13(H3N2)
	**AA**	99% A/sw/IN/A01202867/11(H3N2)	97% A/sw/ON/107-22/12(H3N2)	99% A/sw/MB/D0218/13(H3N2)
M	**NA**	99% A/sw/IN/A01260155/12(H3N2)	99% A/sw/MI/A01104086/15(mixed)	99% A/sw/MB/D0208/13(H3N2)
M1	**AA**	99% A/sw/KS/A01377287/14(H1N2)	100% A/sw/KS/A01377287/14(H1N2)	100% A/CA/07/09(H1N1)
M2	**AA**	99% A/ON/3620/10(H1N1)	100% A/ON/3620/10(H1N1)	99% A/GA/06/12(H1N1)
NS	**NA**	99% A/sw/IN/A01202867/11(H3N2)	99% A/sw/IN/A01202867/11(H3N2)	99% A/sw/MB/D0181/12(H3N2)
	**AA**	99% A/sw/IN/A01202867/11(H3N2)	99% A/sw/IN/A01202866/11(H3N2)	99% A/sw/IA/13C019/13(H3N2)

gene		**A/SW/ON/G16/2014**
PB2	**NA**[a]	99% A/sw/ON/107-22/12(H3N2)
	**AA**[b]	99% A/sw/IN/A01202867/11(H3N2)
PB1	**NA**	99% A/turkey/ON/FAV-10/11(H3N2)
	**AA**	99% A/sw/IN/A01202867/11(H3N2)
PA	**NA**	99% A/sw/ON/204-76/12(H3N2)
	**AA**	99% A/sw/MI/A01202036/11(H1N1)
HA	**NA**	99% A/sw/ON/105-56/12(H3N2)
	**AA**	98% A/sw/ON/105-56/12(H3N2)
NP	**NA**	99% A/sw/ON/204-76/12(H3N2)
	**AA**	99% A/sw/AB/OTH-33-14/09(H1N1)
NA	**NA**	98% A/sw/ON/107-22/12(H3N2)
	**AA**	97% A/sw/ON/107-22/12(H3N2)
M	**NA**	99% A/sw/MI/A01104086/15(mixed)
M1	**AA**	100% A/sw/KS/A01377287/14(H1N2)
M2	**AA**	100% A/ON/3620/10(H1N1)
NS	**NA**	99% A/sw/ON/114-13/12(H3N2)
	**AA**	100% A/sw/ON/114-13/12(H3N2)

Table S2. Amino acid sequence substitutions observed in the HA1 of 13 H3N2 viruses isolated from Canadian swine.

**Antigenic site**	***C***	***C***	***C***	***E***	***E***	***E***	***E***	***E***	***A***	***A***	***A***	***A***	***A***	***A***	***A***	***A***	***A***
**Amino acid**	G	G	N	H	D	E	S	Y	T	Q	N	N	A	D	R	S	R
**Amino acid position**	49	50	53	75	78	83	91	94	121	122	124	126	131	133	137	138	140
**Virus**																	
G1-A/SW/ON/120-55/12/H3N2		R				G									Y	A	
A/SW/ON/33853/05/H3N2		R									S				Y	A	
G2-A/SW/ON/104-25/12/H3N2											S						
G3-A/SW/ON/103-18/11/H3N2		R					N	H			S				Y	A	
A/SW/ON/G10/14/H3N2				N	E					P	S				Y	A	
A/SW/ON/G3/14/H3N2								H						N			K
A/SW/ON/G13/14/H3N2								H						N			K
A/SW/ON/G11/14/H3N2						K											
A/SW/ON/G16/14/H3N2						K											
A/SW/ON/G12/14/H3N2						K											
A/SW/ON/G14/14/H3N2						K											
A/SW/MB/G7/14/H3N2																	
A/SW/SK/G8/14/H3N2														N			
A/SW/MB/G5/14/H3N2		R							A	H	S	D		N	T	A	K
A/SW/ON/G15/14/H3N2		R	S			K			N		S				I	A	
A/SW/MB/G1/14/H3N2	D	E				K					S				Y		
A/SW/AB/G9/14/H3N2		I									S		S	G	Y	T	M

Table S2. *Cont.*

Antigenic site	*A*	*A*	*B*	*B*	*B*	*B*	*B*	*B*	*B*	*B*	*B*	*B*	*D*	*D*	*D*	*B*	*C*	*C*
**Amino acid**	D	N	Y	H	N	H	K	G	E	S	Q	S	R	V	I	N	P	D
**Amino acid position**	144	145	155	156	158	159	160	186	189	193	197	199	201	223	242	246	273	275
**Virus**																		
G1-A/SW/ON/120-55/12/H3N2	V			N		Y			K	N								G
A/SW/ON/33853/05/H3N2	V		H	N	D	Y			R	N								G
G2-A/SW/ON/104-25/12/H3N2					G												S	
G3-A/SW/ON/103-18/11/H3N2	V								K		E			G				
A/SW/ON/G10/14/H3N2	V			N		Y			K	N								G
A/SW/ON/G3/14/H3N2	G		H		G												S	
A/SW/ON/G13/14/H3N2	G		H		G												S	
A/SW/ON/G11/14/H3N2					S													
A/SW/ON/G16/14/H3N2					S													
A/SW/ON/G12/14/H3N2					S												S	
A/SW/ON/G14/14/H3N2					S													
A/SW/MB/G7/14/H3N2					S													
A/SW/SK/G8/14/H3N2					G			S										
A/SW/MB/G5/14/H3N2	V				K		Q		N									G
A/SW/ON/G15/14/H3N2	N	K				N			K	Y		P						
A/SW/MB/G1/14/H3N2	G	K		N		S			K				G					G
A/SW/AB/G9/14/H3N2	A	I	H	K		G			N						V	V		G

Table S3. Unique changes exhibited at antigenic site B at amino acid position 155-160, compared to prototype cluster IV virus.

**Viruses**	***155-160 aa***
A/SW/ON33853/05/H3N2	HNLDYK
A/SW/MB/G1/14/H3N2	**Y**NL**NS**K
A/SW/ON/G3/14/H3N2	HNL**GH**K
A/SW/MB/G5/14/H3N2	**YH**L**KHQ**
A/SW/MB/G7/14/H3N2	**YH**L**SHL**
A/SW/SK/G8/14/H3N2	**YH**L**GH**K
A/SW/AB/G9/14/H3N2	H**K**L**NG**K
A/SW/ON/G10/14/H3N2	**Y**NL**N**YK
A/SW/ON/G11/14/H3N2	**YH**L**SHL**
A/SW/ON/G12/14/H3N2	**YH**L**SHL**
A/SW/ON/G13/14/H3N2	HNL**GH**K
A/SW/ON/G14/14/H3N2	**YH**L**SHL**
A/SW/ON/G15/14/H3N2	**YH**L**NN**K
A/SW/ON/G16/14/H3N2	**YH**L**SHL**

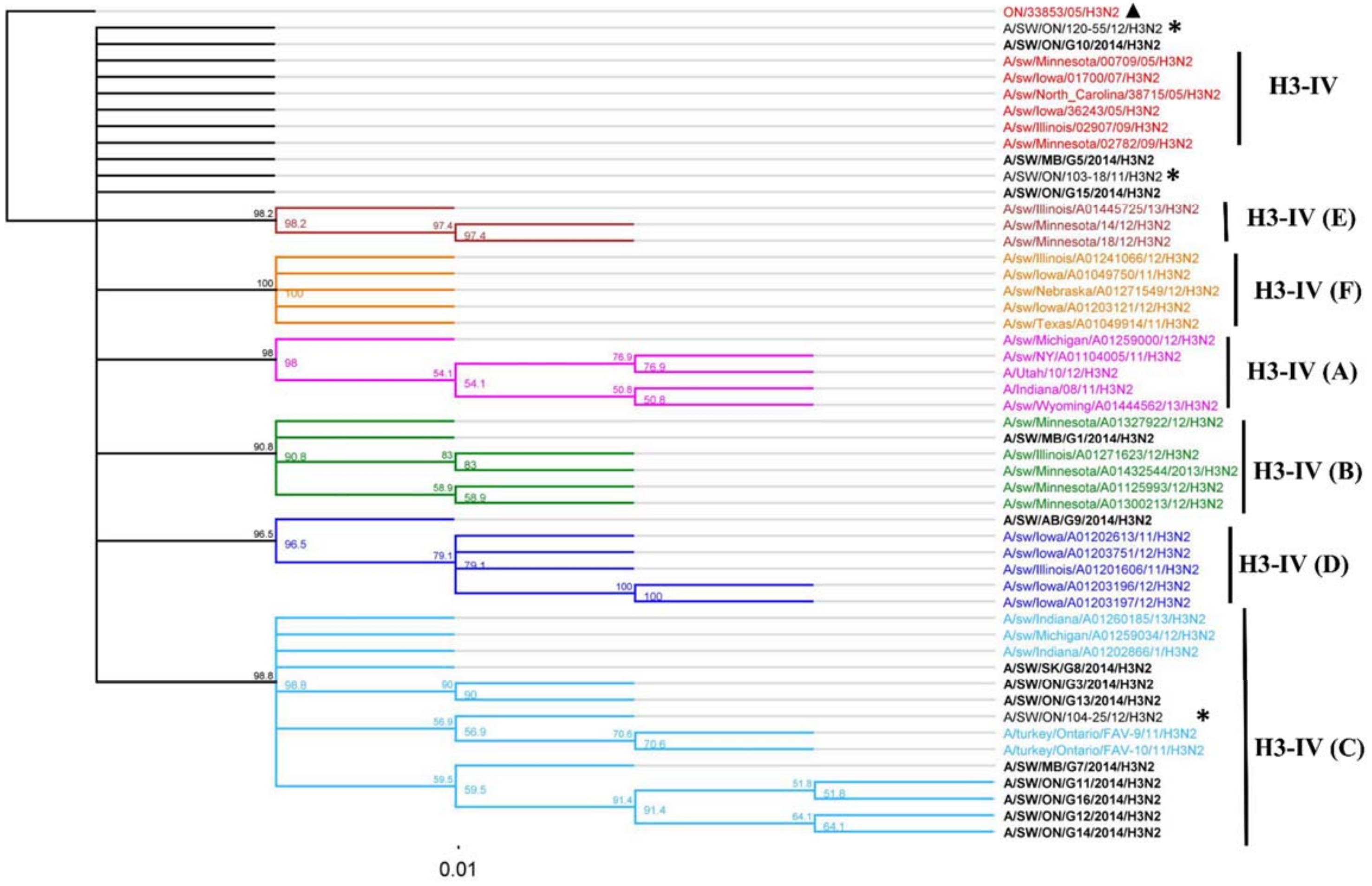

Figure S4. The H3N2 HA phylogeny of Cluster IV from A to F (indicated on right), based on amino acid (*aa*) sequences of the HA1 region. The three is based on Neighbor Joining method with 1,000 bootstraps, and is produced in PAUP. Thirteen Canadian H3N2 viruses isolated during 2014 from swine are labeled in black. Ontario viruses isolated in 2011-2012 are indicated by an asterisk. The 2005 Ontario virus representative of Cluster IV is marked with ▲.

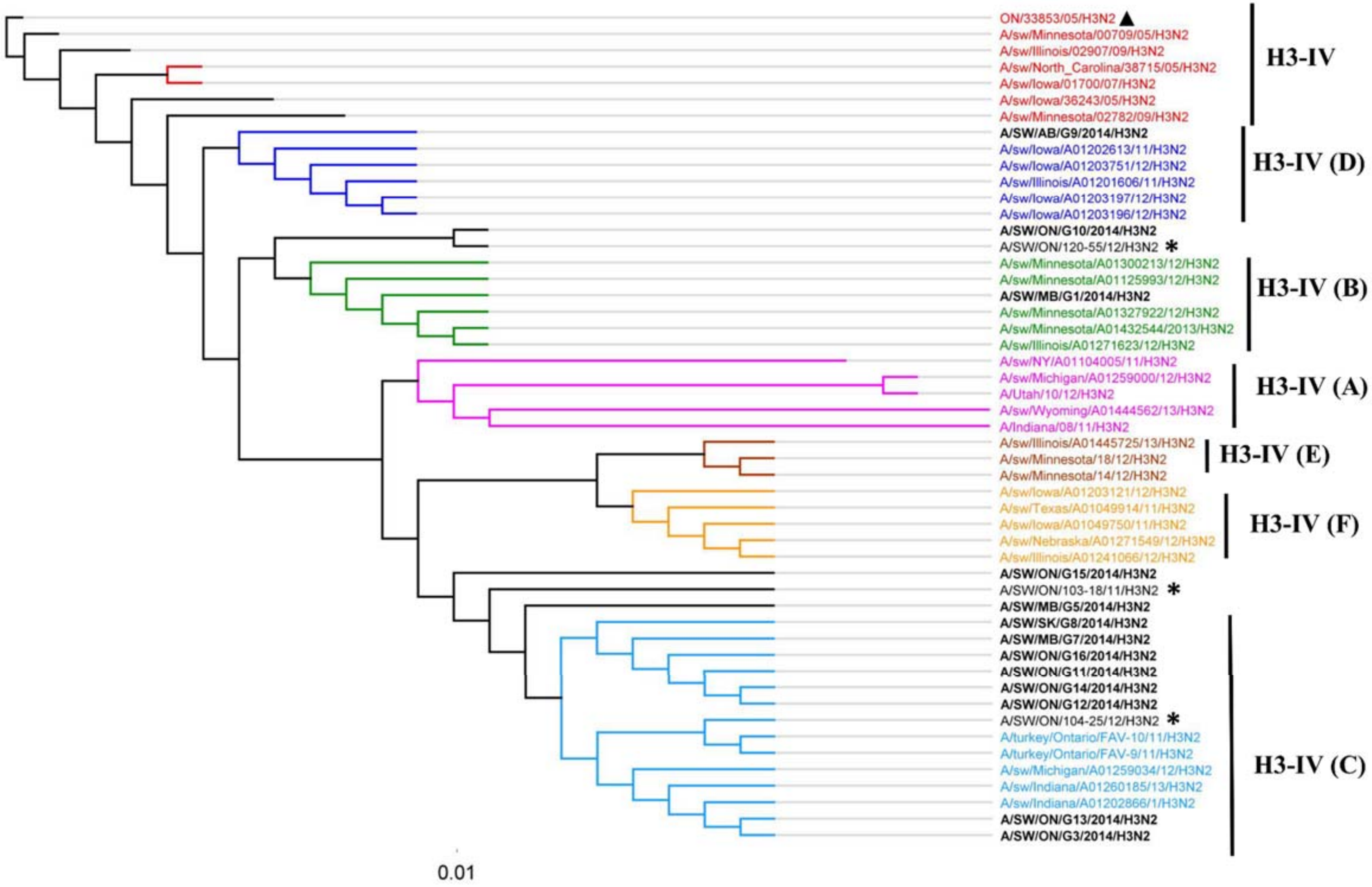

Figure S5. The H3N2 HA phylogeny of Cluster IV from A to F (indicated on right), based on amino acid (*aa*) sequences of the HA1 region. The three is based on Bayesian analysis, and is produced in MrBayes. Thirteen Canadian H3N2 viruses isolated during 2014 from swine are labeled in black. Ontario viruses isolated in 2011-2012 are indicated by an asterisk. The 2005 Ontario virus representative of Cluster IV is marked with ▲.

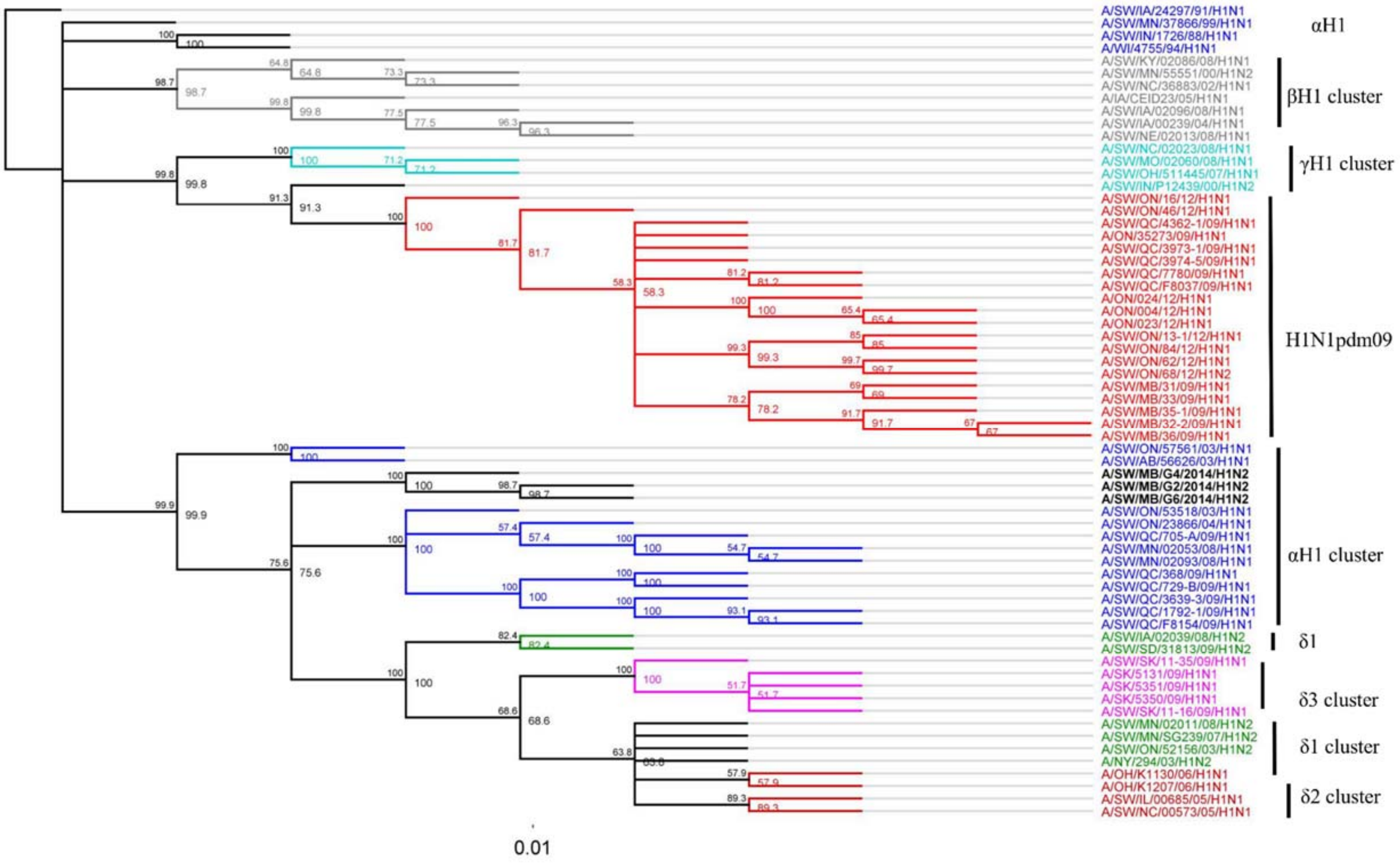

Figure S6. Phylogenetic analysis of the full hemagglutinin (HA) gene of 3 Canadian H1N2 viruses isolated from swine in 2014 (black) based on nucleotide sequences. IAV in swine (IAV-S) H1α (blue), β (gray), γ (turquoise), δ1 (green), δ2 (brown), δ3 (pink) clusters and pandemic (H1N1pdm09) (red) have been included in this analysis. The three is based on Neighbor Joining method with 1,000 bootstraps, and analysis is conducted in PAUP.

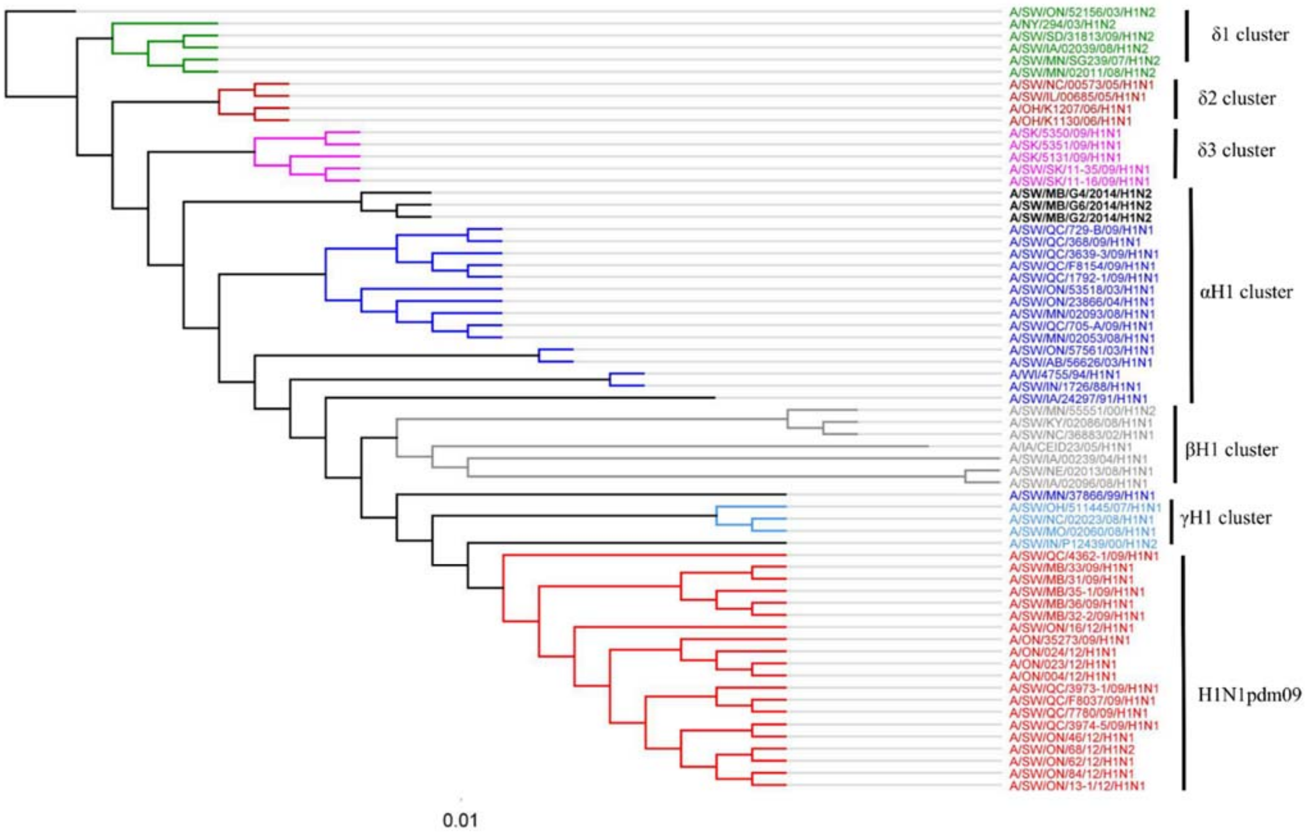

Figure S7. Phylogenetic analysis of the full hemagglutinin (HA) gene of 3 Canadian H1N2 viruses isolated from swine in 2014 (black) based on nucleotide sequences. IAV in swine (IAV-S) H1α (blue), β (gray), γ (turquoise), δ1 (green), δ2 (brown), δ3 (pink) clusters and pandemic (H1N1pdm09) (red) have been included in this analysis. The three is produced on the basis of Bayesian analysis conducted in MrBayes.

© 2017 by the authors. Licensee MDPI, Basel, Switzerland. This article is an open access article distributed under the terms and conditions of the Creative Commons by Attribution (CC-BY) license (http://creativecommons.org/licenses/by/4.0/).
